# Magnetic resonance imaging of the brain in survivors of childhood acute lymphoblastic leukemia

**DOI:** 10.3892/ol.2012.1072

**Published:** 2012-12-12

**Authors:** MOHAMED AHMED BADR, TAMER HASAN HASSAN, KHALED MOHAMED EL-GERBY, MOHAMED EL-SAYED LAMEY

**Affiliations:** 1 Departments of Pediatrics, Zagazig University, Zagazig, Egypt; 2 Radiodiagnosis, Zagazig University, Zagazig, Egypt

**Keywords:** acute lymphoblastic leukemia, magnetic resonance imaging, brain, survivors

## Abstract

The issue of delayed neurological damage as a result of treatment is becoming increasingly important now that an increased number of children survive treatment for acute lymphoblastic leukemia (ALL). Following modification of the treatment protocols, severe symptomatic late effects are rare, and most adverse effects are detected by sensitive imaging methods such as magnetic resonance imaging (MRI) or by neuropsychological testing. In this study we aimed to determine the prevalence and characteristics of late central nervous system (CNS) damage by MRI and clinical examination in children treated for ALL. A cross-sectional study was carried out at the pediatric oncology unit of Zagazig University, Egypt, and included 25 patients who were consecutively enrolled and treated according to the modified Children’s Cancer Group (CCG) 1991 protocol for standard risk ALL and the modified CCG 1961 protocol for high-risk ALL and who had survived more than 5 years from the diagnosis. All relevant data were collected from patients’ medical records; particularly the data concerning the initial clinical presentation and initial brain imaging. All patients were subjected to thorough history and full physical examination with special emphasis on the neurological system. MRI of the brain was performed for all patients. The mean age of patients was 6.9±3.04 years at diagnosis and was 12.9±3.2 years at the time of study. The patients comprised 14 boys and 11 girls. Abnormal MRI findings were detected in six patients (24%). They were in the form of leukoencephalopathy in two patients (8%), brain atrophy in two patients (8%), old infarct in one patient (4%) and old hemorrhage in one patient (4%). The number of abnormal MRI findings was significantly higher in high-risk patients, patients who had CNS manifestations at diagnosis and patients who had received cranial irradiation. We concluded that cranial irradiation is associated with higher incidence of MRI changes in children treated for ALL. Limitation of cranial irradiation to selected patients contributed to a lower incidence of neurological complications in our study. MRI is a sensitive radiological tool to detect structural changes in children treated for ALL, even in asymptomatic cases.

## Introduction

Acute lymphoblastic leukemia (ALL) is the most common malignancy in children. It accounts for approximately 25% of all childhood cancers and almost 75% of childhood leukemias. Treatment results in childhood ALL are one of the true success stories of modern clinical oncology with an overall cure rate currently approaching more than 85% in the developed world, mainly through the application of intensive multi-agent chemotherapeutic regimens (1,2).

This therapeutic progress is the result of treatment advances that began with the identification of effective single agent chemotherapy in the late 1940s, followed by development of combination chemotherapy and maintenance chemotherapy in the 1950s and early 1960s and the implementation of effective central nervous system (CNS) preventive therapy in the 1960s and 1970s (3).

CNS-directed therapy is a key contributing factor to improving survival among children with ALL. When cranial radiation was linked to neurocognitive deficits, therapeutic regimens were modified to reduce or eliminate cranial radiation and substituted it with intensified intrathecal and systemic chemotherapy. These CNS-directed therapies could also influence the risk of late neurological outcomes (4).

Neurological complications are common, both during and following completion of therapy (5). Common neurological complications developing after completion of ALL treatment include leukoencephalopathy and neurocognitive defects (6).

Several studies have used magnetic resonance imaging (MRI) to detect neurologic complications in patients treated for ALL. A wide range of results have been reported (7,8).

Hemosiderin and white matter lesions are two of the most common neurological complications found on MRI that may be related to cranial irradiation and intrathecal methotrexate (MTX) therapy in childhood ALL (5).

We aimed to determine the prevalence and characteristics of late CNS damage by MRI and clinical examination in children treated for ALL.

## Materials and methods

### 

#### Patients

This study was carried out at the outpatient clinic of the Pediatric Oncology Unit of Zagazig University Hospital and the MRI Unit of the Radiodiagnosis Department of Zagazig University between September 2010 and August 2011. It included 25 patients who were consecutively enrolled and treated according to the modified Children’s Cancer Group (CCG) 1991 protocol for standard risk ALL and modified CCG 1961 protocol for high-risk ALL and who had survived more than 5 years from the diagnosis. The modified CCG 1991 protocol for standard risk ALL and modified CCG 1961 protocol for high-risk ALL have been applied as a unified protocol in Egypt since 2004.

All relevant data were collected from patients’ medical records, specifically those concerning the initial clinical presentation and initial brain imaging.

All patients were subjected to: i) Thorough history and full physical examination with special emphasis on the neurological system; ii) MRI of the brain using Philips Achieva class II MRI 1.5-T scanner (Philips Medical Systems, Best, The Netherlands) using T1-weighted (T1W) sagittal spin-echo [repetition time (TR), 500 msec; echo time (TE), 15 msec], T2-weighted transverse fast spin-echo (TR, 3,300 msec; TE, 100 msec), GE transverse (TR, 300 msec; TE, 30 msec; flip angle, 30°), and fluid attenuated inversion recovery (FLAIR) coronal (TR, 8,000 msec; TE, 110 msec; T1, 2,400 msec) sequences.

### Definitions of abnormal MRI findings

#### Leukoencephalopathy

Hyperintense white matter abnormalities were graded according to a modification of the system of Wilson *et al*, 1991. Grade I was defined as patchy, mildly increased signal intensity in the periventricular white matter, grade II as moderate changes that extend almost to the gray-white junction, sparing the subcortical U-fibers, and grade 3 as severe changes, confluent from the level of the frontal horns to that of the trigones, with or without involvement of the U-fibers.

##### Brain atrophy

The definition was based on visual evaluation of the width of cortical sulci and the size of the ventricles and was divided into three grades (mild, moderate and severe).

##### Old infarcts

Old infarcts were diagnosed by the detection of brain parenchymal loss pertaining to arterial territory or discrete lesions with hypointensity on T1-weighted images and hyperintensity on T2-weighted images.

##### Old hemorrhages

Old hemorrhages were defined as focal rounded areas of very low signal intensity (attributable to the presence of hemosiderin) detected in any part of the brain.

#### Summary of modified CCG 1991 protocol for standard risk ALL

Patients were eligible for this protocol if they had previously untreated ALL with >25% blasts (L1 or L2 morphology) in bone marrow, age 1–9.99 years and initial WBC <50.000/*μ*l. Patients with CNS disease at diagnosis, overt testicular leukemia, FAB L3 and T-cell ALL (T-ALL) were not eligible.

Patients received induction chemotherapy for one month (i.v. vincristine, p.o. dexamethasone, i.m. L-asparaginase, i.t. MTX and i.t. Ara-C), consolidation therapy for 4 weeks (i.v. vincristine, p.o. 6-mercaptopurine and i.t. MTX), interim maintenance I for 2 months (i.v. vincristine, p.o. dexamethasone, p.o. 6-mercaptopurine, p.o. MTX and i.t. MTX), delayed intensification for 2 months (i.v. vincristine, i.v. doxorubicin, p.o. dexamethasone, i.m. L-asparaginase, i.v. cylophosphamide, p.o. 6-thioguanine, i.v. or s.c. Ara-C and i.t. MTX), interim maintenance II for 2 months (same as interim maintenance I) and maintenance 12-week cycles (i.v. vincristine, p.o. dexamethasone, p.o. 6-mercaptopurine, p.o. MTX and i.t. MTX). Therapy was continued for 2 calendar years for girls and 3 calendar years for boys.

#### Summary of modified CCG 1961 protocol for high-risk ALL

Patients were eligible for this protocol if they had previously untreated ALL with >25% blasts (L1 or L2 morphology) in bone marrow. Patients with FAB L3 were not eligible.

##### A) Standard arm

The standard arm included patients aged 1–9.99 years with initial WBC >50.000/*μ*l, patients aged >10 years with any WBC count, patients with overt testicular leukemia and patients with T-ALL. Patients in the standard arm received induction chemotherapy for one month (same as standard risk plus i.v. doxorubicin), consolidation therapy for 5 weeks (i.v. cylophosphamide, p.o. 6-mercaptopurine, i.v. or s.c. Ara-C and i.t. MTX), interim maintenance I for 2 months (p.o. 6-mercaptopurine, p.o. MTX and i.t. MTX), delayed intensification for 2 months (i.v. vincristine, i.v. doxorubicin, p.o. dexamethasone, i.m. L-asparaginase, i.v. cylophosphamide, p.o. 6-thioguanine, i.v. or s.c. Ara-C and i.t. MTX), interim maintenance II for 2 months (same as interim maintenance I) and maintenance 12-week cycles (same as standard risk treatment). Therapy was continued for 2 calendar years for girls and 3 calendar years for boys.

##### B) Augmented arm

The augmented arm included patients with CNS disease at diagnosis and patients with poor response at day 14 (both standard and high risk). Patients received induction chemotherapy for one month (same as standard risk treatment plus i.v. doxorubicin), consolidation for 9 weeks (i.v. vincristine, i.v. cylophosphamide, i.m. L-asparaginase, p.o. 6-mercaptopurine, i.v. or s.c. Ara-C and i.t. MTX plus cranial radiotherapy 18 Gy for patients without CNS disease at diagnosis and 24 Gy for those with CNS disease at diagnosis), interim maintenance I and II, each for 2 months (i.v. vincristine, i.v. MTX, i.m. L-asparaginase and i.t. MTX), delayed intensification I and II for 8 weeks (i.v. vincristine, i.v. doxorubicin, p.o. dexamethasone, i.m. L-asparaginase, i.v. cylophosphamide, p.o. 6-thioguanine, i.v. or s.c. Ara-C and i.t. MTX) and maintenance 12-week cycles (same as standard risk treatment). Therapy was continued for 2 calendar years for girls and 3 calendar years for boys.

#### Ethics

The study was performed in accordance with ethical standards and with the Helsinki Declaration of 1964, as revised in 2000. The study was approved by the local ethics committee and informed consent was obtained from the study participants.

#### Statistical analysis

Data were checked, entered and analyzed using SPSS version 11. Data are expressed as the mean ± standard deviation for quantitative variables, and as a number and percentage for qualitative ones. Paired t-test and Chi-square (χ^2^) tests were used when appropriate. P<0.05 was considered to indicate a statistically significant result.

## Results

### 

#### Patient characteristics

The demographic, clinical and laboratory data of the patients are listed in Table I.

#### MRI findings of patients

Abnormal MRI findings were detected in six patients (24%). The abnormalities were in the form of leukoencephalopathy in two patients (one had grade III and the other had grade I) (8%), brain atrophy in two patients (8%), old infarct in one patient (4%) and old hemorrhage in one patient (4%) (Table II).

#### Correlation between MRI findings and demographic data of patients

There was no significant correlation between MRI findings and the age and gender of patients (P=0.37 and P=0.89, respectively).

#### Correlation between MRI findings and immunophenotyping of leukemic cells

There was no significant correlation between MRI findings and immunophenotyping (P=0.75).

#### Correlation between MRI findings and risk group of patients

There was a significant correlation between MRI findings and risk group, where abnormal MRI findings were significantly higher in high-risk patients (P=0.04; Table III).

#### Correlation between MRI findings and treatment protocol

There was a significant correlation between MRI findings and treatment protocol where abnormal MRI findings were significantly higher in patients who received CCG high-risk (augmented arm) protocol (P<0.001; Table IV).

#### Correlation between MRI findings and CNS manifestations at diagnosis

There was a significant correlation between MRI findings and CNS manifestations at diagnosis where abnormal MRI findings were significantly higher in patients with CNS manifestations at diagnosis (Table V).

#### Correlation between MRI findings and cranial irradiation

There was a significant correlation between MRI findings and cranial irradiation where abnormal MRI findings were significantly higher in patients who received cranial irradiation (P<0.001; Table VI).

#### Correlation between MRI findings and late neurological complications

There was no significant correlation between MRI findings and late neurological complications (P=0.37).

## Discussion

Currently, the overall long-term survival rate in ALL is approximately 70%, although for individual patients this varies from 40–90%, depending on prognostic features at diagnosis and early response to therapy (9,10). Previously, however, CNS relapse occurred in at least 50% of patients (11).

This phenomenon led to the introduction of CNS prophylaxis for children with ALL. The most commonly used method in the 1970s and 1980s involved cranial irradiation, originally at a dose of 24 Gy but later at 18 Gy, and intrathecal chemotherapy with MTX (12). This treatment reduces the rate of isolated CNS relapse to 5–10% but is associated with various forms of damage to normal brain tissue, including leukoencephalopathy, mineralizing microangiopathy (MMA), and the development of secondary tumors (11).

Cranial irradiation appears to be a notable cause of long-term neuropsychological impairment (13). Protocols have used risk stratification to avoid cranial irradiation in children with standard and intermediate-risk ALL (14,15) and reduced the dose to 12 Gy in those with high-risk ALL, without compromising event-free survival (3).

**Figure 1.**
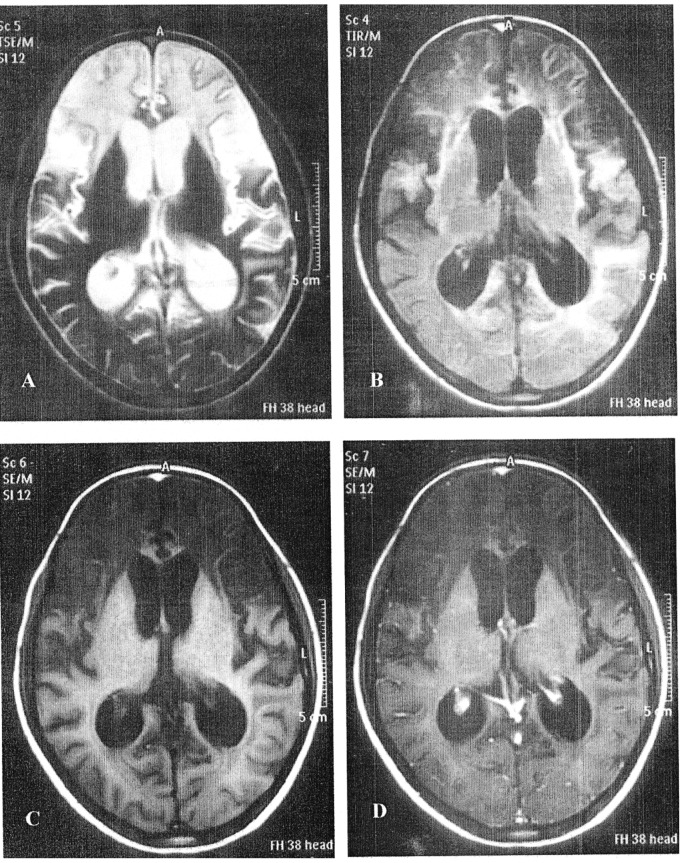
Magnetic resonance imaging of the brain of a 13-year-old boy previously treated with cranial irradiation. Diffuse bilateral confluent cortical and subcortical areas of abnormal attenuation are visible in both cerebral hemispheres, mainly in the frontal and parietal areas. High signal intensity was observed on (A) T2W1 and (B) FLAIR and low signal intensity was observed on T1W1 (C) without contrast and (D) with intravenous contrast. The images are consistent with grade III leukoencephalopathy. FLAIR, fluid attenuated inversion recovery; T2W, T2-weighted; T1W, T1-weighted.

In our study, abnormal MRI findings were detected in six patients (24%). They were in the form of leukoencephalopathy in two patients (one had grade III and the other had grade I) (8%), brain atrophy in two patients (8%), old infarct in one patient (4%) and old hemorrhage in one patient (4%). The brain MRI of a 13-year-old boy with grade III leukoencephalopathy is shown in Fig. 1.

Our results are similar to those reported by Ficek *et al*(16) where white matter changes were detected by MRI in three (11%) out of 45 ALL survivors treated between 1994 and 2002 and examined for 6–12 years following treatment. All children with MRI abnormalities received CRT.

Pääkkö *et al*(7) carried out a prospective study on 33 children with ALL and observed high-intensity white matter changes by MRI in 3 children (9%) who received chemotherapy only.

Aytaç *et al*(17) reviewed the data of 256 children with ALL who were admitted to the Pediatric Hematology Unit of Hacettepe University, Turkey, between March 1991 and May 2005 and who were eligible for and treated according to the St. Jude Total XI and XIII protocols. Abnormal MRI findings were reported in only one out of five patients, for whom MRI was performed following cessation of treatment. The abnormalities were in the form of an increase in white matter intensity.

Chan *et al*(5) evaluated the brains of 42 patients diagnosed more than 5 years previously. Forty of the ALL patients had been treated with cranial irradiation (at least 18 Gy) and intrathecal MTX, as well as systemic chemotherapy, and two had been treated with intrathecal MTX and systemic chemotherapy but had not received cranial irradiation. Lesions consistent with old hemorrhage were detected in 23 (55%) of the ALL patients, white matter abnormalities were found in two patients (5%) while old infarcts were observed in four patients (10%). Lesions were observed in all 40 patients who underwent cranial irradiation.

Our results showed that there was no significant correlation between MRI findings and age, gender or immunophenotyping of leukemic cells.

Conversely, Pääkkö *et al*(7) reported that children with white matter changes were significantly younger than those with normal MRI (mean age 2.8 vs. 7.4 years).

In our study, cranial irradiation was administered to only four patients who received the augmented arm of the CCG high-risk protocol. Three of them were assigned to this arm based on their initial CNS infiltration and one was assigned based on his poor response to the standard arm of this high-risk protocol.

In our study, there was a significant (P<0.001) correlation between MRI findings and cranial irradiation. All patients who received cranial irradiation developed abnormal MRI findings while only two out of 21 (9.5%) who received systemic chemotherapy and intrathecal methotrexate without cranial irradiation developed abnormal MRI findings. Our results augment and support the idea that cranial irradiation should be avoided in patients with standard risk criteria. In support of this theory, Chan *et al*(5) and Ficek *et al*(16) reported that all abnormal MRI lesions were observed in patients who underwent cranial irradiation.

Radiation-induced brain toxicity was explained by Kim *et al*(18) who found that radiation injures the supportive tissues and neurogenic microenvironment of the nervous system and leads to neuronal loss or damage. Oxygen-free-radical damage and altered cytokine responses may influence the development of late delayed damage. Glial and neuronal stem-cell damage may result in a progressive demyelination and/or neuronal cell loss.

In our study, there was a significant correlation between MRI findings and risk group (P=0.04) where five out of six patients who developed abnormal MRI findings belonged to the high-risk group and only one belonged to the standard risk group. Also, there was a significant (P<0.001) correlation between MRI findings and treatment protocol where the four patients (100%) who received the CCG high-risk augmented arm protocol developed abnormal MRI findings while one out of 15 (6.7%) who received the CCG high-risk standard arm protocol developed abnormal MRI findings and one out of six (16.7%) who received the CCG standard risk protocol developed abnormal MRI findings. This finding can be attributed to the fact that patients who received the CCG high-risk augmented arm underwent cranial irradiation. In our study, there was a significant correlation (P= 0.01) between MRI findings and CNS infiltration at diagnosis where all patients with CNS infiltration at initial diagnosis developed abnormal MRI findings and only three out of 22 (13.6%) without CNS infiltration at diagnosis developed abnormal MRI findings. This can once more be attributed to patients with CNS infiltration at initial diagnosis receiving the CCG high-risk augmented arm protocol and undergoing cranial irradiation.

In our study, only two of our patients developed late neurological complications. One patient developed recurrent seizures and proved to have epilepsy. This patient belonged to the high-risk group and received the CCG high-risk standard arm protocol. He did not receive cranial irradiation and his MRI examination was completely normal. The second patient developed recurrent seizures, abnormal behavioral changes and severe cognitive changes. This patient belonged to the high-risk group and received CCG high-risk augmented arm protocol. He received cranial irradiation and he had grade III leukoencephalopathy by MRI examination.

The lower incidence (8%) of late neurological complications in our study can be explained by the fact that most (84%) of our patients did not receive cranial irradiation. Cranial irradiation is reserved for patients with CNS infiltration at initial diagnosis and for those with poor response to the standard risk protocol and the standard arm of the high-risk protocol. It can also be explained by the fact that 60% of our patients belonged to the standard risk group who received tolerable doses of chemotherapeutics.

In our study, there was no significant correlation between MRI findings and late neurological complications. This can be attributed to the small number of patients with late neurological complications.

We conclude that cranial irradiation is associated with higher incidence of MRI changes in children treated for ALL. Limitation of cranial irradiation to selected patients contributed to the lower incidence of neurological complications in our study. MRI is a sensitive radiological tool to detect structural changes in children treated for ALL, even in asymptomatic cases.

## Figures and Tables

**Table I.** Demographic, clinical and laboratory data of patients.

Parameter	n	%
Age at diagnosis (years)		
Mean ± SD	6.9±3.04	
Range	2.5–13	
Age at study (years)		
Mean ± SD	12.9±3.2	
Range	8.5–20	
Gender		
Male	14	56.0
Female	11	44.0
Risk		
SR	15	60.0
HR	10	40.0
Protocol of treatment		
CCG-SR	15	60.0
CCG-HR-SA	6	24.0
CCG-HR-AA	4	16.0
Immunophenotyping		
Precursor B-ALL	22	88.0
T-ALL	3	12.0
CNS manifestations at diagnosis		
Yes	3	12.0
No	22	88.0
Cranial irradiation		
Yes	4	16.0
No	21	84.0
Late neurological complications		
None	23	92.0
Epilepsy	1	4.0
Cognitive changes, behavioral changes, epilepsy	1	4.0

SR, standard risk; HR, high risk; CCG-SR, Children’s Cancer Group - standard risk; CCG-HR-SA: Children’s Cancer Group - high risk, standard arm; CCG-HR-AA, Children’s Cancer Group - high risk, augmented arm; ALL, acute lymphoblastic leukemia; CNS, central nervous system.

**Table II.** MRI findings of patients (n=25).

MRI findings	n	%
Normal	19	76.0
Leukoencephalopathy	2	8.0
Brain atrophy	2	8.0
Old infarct	1	4.0
Old hemorrhage	1	4.0

MRI, magnetic resonance imaging.

**Table III.** Correlation between MRI findings and risk group.

	MRI normal (n=19)		MRI abnormal (n=6)			
Risk	n	%	n	%	χ^2^	P-value
SR (n=15)	14	73.7	1	16.7	4.03	0.04^a^
HR (n=10)	5	26.3	5	83.3		

MRI, magnetic resonance imaging; SR, standard risk; HR, high risk.

a Statistically significant.

**Table IV.** Correlation between MRI findings and treatment protocol.

	MRI normal (n=19)		MRI abnormal (n=6)			
Protocol	n	%	n	%	χ^2^	P-value
CCG-SR (n=15)	14	73.7	1	16.7		
CCG-HR-SA (n=6)	5	26.3	1	16.7	15.31	<0.001^a^
CCG-HR-AA (n=4)	0	0.0	4	66.6		

MRI, magnetic resonance imaging; CCG-SR, Children’s Cancer Group - standard risk; CCG-HR-SA, Children’s Cancer Group - high risk (standard arm); CCG-HR-AA, Children’s Cancer Group - high risk (augmented arm).

a Significant.

**Table V.** Correlation between MRI findings and CNS manifestations at diagnosis.

	MRI normal (n=19)		MRI abnormal (n=6)			
CNS manifestations at diagnosis	n	%	n	%	χ^2^	P-value
Yes (n=3)	0	0.0	3	50.0	6.58	0.01^a^
No (n=22)	19	100.0	3	50.0		

MRI, magnetic resonance imaging; CNS, central nervous system.

a Statistically significant.

**Table VI.** Correlation between MRI findings and cranial irradiation.

	MRI normal (n=19)		MRI abnormal (n=6)			
Cranial irradiation	n	%	n	%	χ^2^	P-value
Yes (n=4)	0	0.0	4	66.7	10.53	<0.001^a^
No (n=21)	19	100.0	2	33.3		

MRI, magnetic resonance imaging.

a Significant.
